# Draft Genome Assembly of the Freshwater Apex Predator Wels Catfish (*Silurus glanis*) Using Linked-Read Sequencing

**DOI:** 10.1534/g3.120.401711

**Published:** 2020-09-11

**Authors:** Mikhail Yu. Ozerov, Martin Flajšhans, Kristina Noreikiene, Anti Vasemägi, Riho Gross

**Affiliations:** * Department of Aquatic Resources, Institute of Freshwater Research, Swedish University of Agricultural Sciences, Drottningholm, 17893, Sweden; † Biodiversity Unit, University of Turku, 20014, Finland; ‡ Department of Biology, University of Turku, 20014, Finland; § South Bohemian Research Center of Aquaculture and Biodiversity of Hydrocenoses, Faculty of Fisheries and Protection of Waters, University of South Bohemia in České Budějovice, 38925 Vodňany, Czech Republic; ** Chair of Aquaculture, Institute of Veterinary Medicine and Animal Sciences, Estonian University of Life Sciences, Tartu, 51014, Estonia

**Keywords:** *Silurus glanis*, wels catfish, whole genome sequencing, *de novo* assembly, 10X Genomics Chromium linked-reads, teleost

## Abstract

The wels catfish (*Silurus glanis*) is one of the largest freshwater fish species in the world. This top predator plays a key role in ecosystem stability, and represents an iconic trophy-fish for recreational fishermen. *S. glanis* is also a highly valued species for its high-quality boneless flesh, and has been cultivated for over 100 years in Eastern and Central Europe. The interest in rearing *S. glanis* continues to grow; the aquaculture production of this species has almost doubled during the last decade. However, despite its high ecological, cultural and economic importance, the available genomic resources for *S. glanis* are very limited. To fulfill this gap we report a *de novo* assembly and annotation of the whole genome sequence of a female *S. glanis*. The linked-read based technology with 10X Genomics Chromium chemistry and Supernova assembler produced a highly continuous draft genome of *S. glanis*: ∼0.8Gb assembly (scaffold *N*_50_ = 3.2 Mb; longest individual scaffold = 13.9 Mb; BUSCO completeness = 84.2%), which included 313.3 Mb of putative repeated sequences. In total, 21,316 protein-coding genes were predicted, of which 96% were annotated functionally from either sequence homology or protein signature searches. The highly continuous genome assembly will be an invaluable resource for aquaculture genomics, genetics, conservation, and breeding research of *S. glanis*.

The wels catfish, also known as the European catfish or sheatfish, *Silurus glanis* (NCBI Taxonomy ID: 94993; Figure 1), is a non-migratory benthopelagic predatory fish that lives mainly in freshwater habitats (large warm lakes and deep, slow-flowing rivers), but also occurs in brackish waters in the Baltic and Black Seas (Frimodt 1995). *S. glanis* is native to the North, Baltic, Black, Caspian and Aral Sea basins; its distribution extends from Germany eastwards through Poland and the Baltic States to Russia, and from Southern Sweden southwards through Eastern European countries to Turkey, Northern Iran and the Aral Sea basin of Kazakhstan and Uzbekistan (Kottelat and Freyhof 2007; Copp *et al.* 2009). Following introduction outside its native range, *S. glanis* has become established in a number of Western and Southern European countries, as well as in Syria and China (Froese and Pauly 2019).

**Figure 1:**
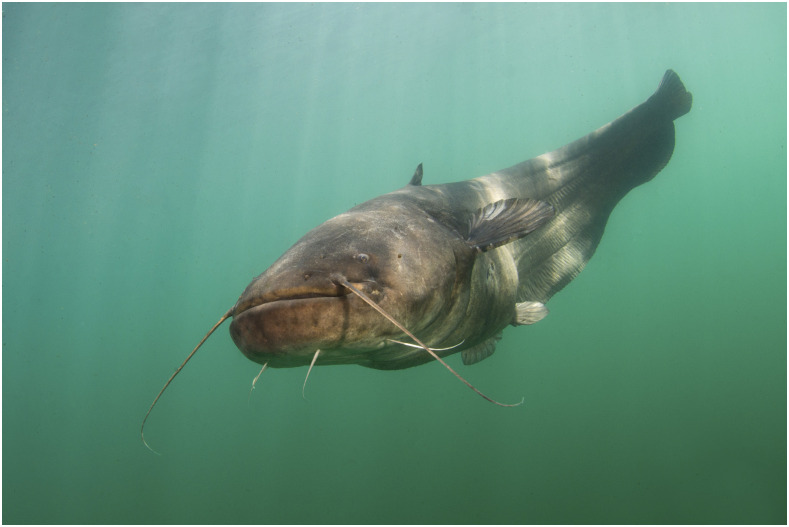
Wels catfish (*Silurus glanis*). Photo by Filip Staes, http://www.fsfotografie.be/.

*S. glanis* belongs to the family Siluridae, a large group of freshwater fishes native to Europe, Asia and Africa. The family includes more than 100 species from 12 genera. There are 18 species in the genus *Silurus*, two of which are native to Europe: *S. glanis* and *S. aristotelis* (Copp *et al.* 2009). *S. glanis* is the largest fish of the order Siluriformes and the largest among European freshwater teleost fishes. Its maximum reported size was 5 m and 306 kg, caught in the River Dniepr (Berg 1949), although the typical length and weight vary from 1.3 to 1.6 m and from 15 to 65 kg, respectively (Frimodt 1995; Alp *et al.* 2011).

*S. glanis* is an ecologically and economically important species. It is the freshwater apex predator, the target of commercial and recreational fisheries, and a rising species in aquaculture (Linhart *et al.* 2002; Vejřík *et al.* 2017; Cucherousset *et al.* 2018). *S. glanis* is particularly popular among European anglers, and its economic importance in many Central and Eastern European countries has increased because the species possesses many characteristics desirable for profitable aquaculture (*e.g.*, its growth rate is among the highest of any fish, and it has tasty white boneless flesh, high carcass yield, and high feed utilization efficiency; Pruszynski and Pistelok 1999; Linhart *et al.* 2002; Jankowska *et al.* 2006; Adamek *et al.* 2015). The aquaculture production of *S. glanis* in Europe, Asia and Africa has grown from 14 tons in 1984 to 2,026 tons in 2018. Currently the largest producers are Uzbekistan, Poland, Hungary, Bulgaria, France, Germany and Czech Republic (FAO 2020).

Despite its high ecological, cultural and economic significance, the available genomic resources for *S. glanis* are very limited. To date, only a mitochondrial genome (Vittas *et al.* 2011) and a few gene sequences of *S. glanis* are available in the NCBI database (https://www.ncbi.nlm.nih.gov/). There are a number of genome assemblies of other Siluriformes (Liu *et al.* 2016; Gong *et al.* 2018; Kim *et al.* 2018; Li *et al.* 2018; Zhang *et al.* 2018; Jiang *et al.* 2019), however, these species are evolutionarily rather distant from *S. glanis* (Kappas *et al.* 2016). Therefore, given the growing interest in the aquaculture of *S. glanis*, there is a strong need for improving the databases of its genetic resources and for developing novel genetic tools to increase the efficiency of breeding programs. Moreover, given that the fisheries based on wild-caught *S. glanis* exceed aquaculture production (FAO 2020), genomic information is essential for developing sustainable conservation strategies for wild populations.

To assemble the *S. glanis* genome, we used the 10X Genomics Chromium technology, a genomic library preparation technique designed to build accurate and highly continuous assemblies from short sequencing reads. In brief, the 10X Genomics library preparation technology incorporates unique molecular barcodes into individual high molecular weight DNA molecules, after which genomic libraries undergo standard Illumina short-read sequencing (Zheng *et al.* 2016). The construction of highly continuous scaffolds is achieved by using these barcodes in a phased assembly strategy algorithm implemented in the Supernova software to tag short reads that come from the same long DNA fragment (known as linked-reads; Weisenfeld *et al.* 2017).

Here, we report a high-quality, highly continuous, and nearly complete assembly of the *S. glanis* genome generated using 10X Genomics linked-read sequencing. This resource will serve as a backbone for aquaculture genomics, genetics, conservation, and breeding research of *S. glanis* and other fish species of the Siluridae family.

## Materials And Methods

### Ethics statement

The requirements outlined in the Annex III (Requirements for establishments and for the care and accommodation of animals) and Annex IV (Methods of killing animals) Section B point 11 of the “Directive 2010/63/EU of the European parliament and of the council of 22 September 2010 on the protection of animals used for scientific purpose” were fully met. The authors have followed the principles of the 3Rs (Replacement, Reduction and Refinement) and have involved the minimum number of animals to produce statistically reproducible results.

### Samples, library preparation and sequencing

The sample tissues were obtained from one 160-day-old *S. glanis* female (weight 346 g, total length 393 mm) of the Hodonin strain, collected in the experimental aquaculture facilities of the Chair of Aquaculture, Estonian University of Life Sciences (Tartu, Estonia). The Hodonin strain originated from the Danube river drainage, and the broodstock are maintained at the Research Institute of Fish Culture and Hydrobiology (RIFCH) of the Faculty of Fisheries and Protection of Waters, University of South Bohemia (Vodňany, Czech Republic). The specimen was killed by an overdose of 2-phenoxyethanol before sampling. A blood sample was collected from the caudal vein into a 2 ml sterile vacuum blood collection tube containing 3.6 mg K_2_EDTA (VACUTEST KIMA, Italy) using a sterile needle with a safety device (21G × 1.5”, VACUTEST KIMA, Italy). The tube was kept on ice during transport to the laboratory, and then stored at +4° until DNA isolation. For transcriptome characterization, tissues from eleven organs of the same individual (barbel, whole eye, fin, gill, gonad (ovary), heart, kidney, muscle, liver, spleen and swim bladder) were dissected, snap frozen in liquid nitrogen and kept at -80° until RNA isolation.

High molecular weight genomic DNA (gDNA) was isolated from blood using the MagAttract HMW DNA Kit (Qiagen, Halden, Germany) according to manufacturer’s instructions, with a few modifications. As fish red blood cells contain a nucleus, we used only 3 µl of buffered blood (instead of the recommended 200 µl). In addition, to avoid fragmentation of high molecular weight DNA, vortexing and mixing procedures were very brief and gentle during the DNA isolation procedure. Total gDNA was eluted in 80 µl of buffer AE (Qiagen, Halden, Germany). The quantity of gDNA was measured by Qubit Fluorometric Quantitation (ThermoFisher), and the average length of the gDNA fragments was determined by the FEMTOPule using the Femto gDNA 165Kb FP-1002 kit (Agilent Technologies). The average estimated fragment size of gDNA was > 65 Kb. Whole genome sequencing libraries were prepared from 0.6 ng DNA using the Chromium Genome Library preparation kit (cat# 120257/58/61/62) according to the manufacturer’s protocol (#CG00043 Chromium Genome Reagent Kit v2 User Guide), and 0.75 ng of template gDNA was loaded on a Chromium Genome Chip. The library was sequenced on two lanes of an Illumina HiSeq X sequencer in rapid run mode, using paired-end sequencing (2 × 150 bp) to generate 655.87 M linked-reads, with a mean read length of 139.5 bp after trimming. The weighted mean molecule size was estimated as 37.8 Kb, and the mean read coverage was ∼102×. The WGS library preparation and sequencing was performed by the SNP&SEQ Technology Platform (Uppsala, Sweden).

Total RNA was extracted from eleven pulverized frozen tissues using a NucleoSpin RNA extraction kit (MACHEREY-NAGEL, Duren, Germany) according to the manufacturer’s guidelines. The concentration and integrity of the extracted RNA samples were measured with TapeStation 2200 (Agilent Technologies). Prior the preparation of sequencing libraries, the quality of the RNA samples was ensured using Bioanalyzer 2100 (Agilent Technologies) electrophoresis, and sample concentration was measured with a Qubit Fluorometric Quantitation (Life Technologies). The RNA integrity number scores (RIN) for all samples were high, ranging from 8.2 to 10.0 (mean = 9.5). The libraries were prepared from 100 ng of total RNA according to the Illumina TruSeq Stranded mRNA Sample Preparation Guide (part no. 15031047) to generate libraries with an insert size of 300-bp. The libraries were sequenced using an Illumina HiSeq 3000 (2 × 75 bp configuration, single lane) at the Finnish Functional Genomics Centre (Turku, Finland).

### Estimation of the genome metrics based on raw reads

Jellyfish v.2.2.6 (Marçais and Kingsford 2011) was applied to generate k-mer counting and frequency distributions of 17-, 21- and 25-mers for quality and barcode trimmed Illumina reads (jellyfish histo -h 3000000). Genome size, heterozygosity and repeat content were estimated based on the generated histograms using GenomeScope (Vurture *et al.* 2017; high frequency k-mer cutoff = 10,000) and findGSE (Sun *et al.* 2018).

### de novo genome assembly

The Supernova v.2.1.1 assembler (Weisenfeld *et al.* 2017) was used to assemble the linked-reads data on a 28 core and 240 Gb RAM CSC – IT Center for Science cPouta virtual private server, based on Intel Xeon CPU E5-2680 v.4 2.4 GHz processors. The Supernova run was performed with default parameters, except maximum reads (–maxreads) was set for 386 million input reads to achieve 56× raw coverage, as suggested in the Supernova protocol.

The 824.5 Mb initial draft genome assembly was presented in pseudohaplotype format and consisted of 31,193 of scaffold sequences (≥ 1 Kb), of which 83.5 Mb (10.1%) represented unknown bases. Duplicated scaffolds (1,359 scaffolds) were removed using GenomeTools sequniq v.1.5.10 (Gremme *et al.* 2013), and only scaffolds with more than 10% of unique sequence were retained (29,812 scaffolds). Further, the redundancy of the genome assembly was reduced in two steps. First, CD-HIT v.4.7 package (Fu *et al.* 2012; Li and Godzik 2006) was applied to cluster all the scaffolds < 2 Mb. When two or more scaffolds showed ≥ 99% similarity, all but the longest scaffold were removed to generate a non-redundant set of < 2 Mb scaffolds. This resulted in removal of 3,568 potentially redundant scaffolds from the assembly. Second, to further reduce potential redundancy, the assembly including the non-redundant set of < 2 Mb scaffolds was self-aligned using LAST v.926 (Kiełbasa *et al.* 2011; identity ≥ 99%, coverage of query sequence ≥ 95%), resulting in exclusion of 540 additional scaffolds. In total, 31.1 Mb (3.9%) were removed from the initial assembly due to potential duplication or redundancy. The potentially redundant scaffolds varied in size from 1 Kb to 62.5 Kb and most of them (84.5%) did not exceed 10 Kb.

The final *S. glanis* genome assembly consisted of 25,703 scaffolds. The assembly was screened for vectors and contaminants using a Kraken v.1.0 (Wood and Salzberg 2014) customized database, which included standard Kraken viral, bacterial, archaeal, plasmid and human databases, additional genomes of *Trypanosoma brucei* (GCF_000210295.1, Jackson *et al.* 2010) and seven fish species (*Cyprinus carpio* GCF_000951615.1, Li J.-T., Chinese Academy of Fishery Science; *Danio rerio* GCF_000002035.6, Howe *et al.* 2013); *Esox lucius* GCF_000721915.3. Rondeau *et al.* 2014); *Lates calcarifer* GCF_001640805.1, Vij *et al.* 2016); *Nothobranchius furzeri* GCF_001465895.1, Senf *et al.* Leibniz Institute for Age Research – Fritz Lipmann; *Oncorhynchus mykiss* GCF_002163495.1 Lien *et al.* Norwegian University of Life Sciences; and *Takifugu rubripes* GCF_000180615.1, Kai *et al.* 2011). In total, 30 and 810 scaffolds were detected as potentially contaminated by unicellular organisms or human DNA, respectively. NCBI’s blastn v.2.9.0 (Boratyn *et al.* 2013) was further applied to align those scaffolds to viral, bacterial, trypanosoma or to human refseq gene sequences. As most of the significant hits did not cover more than 2% of a query sequence, all of the scaffolds were considered as non-contaminated and were thus retained for further analyses.

The quality metrics of the genome assembly were generated using QUAST v.4.5.4 (Gurevich *et al.* 2013). Genome assembly completeness was assessed with BUSCO v.3.0.2b (Simão *et al.* 2015) using a database of ray-finned fishes (*Actinopterygii* obd9) consisting of 4,584 orthologs from 20 fish species. Furthermore, to compare the scaffold-level genome assembly of *S. glanis* in this study with a reported chromosome-level genome assembly of *I. punctatus* (GCA_001660625.1, Liu *et al.* 2016), we performed a synteny analysis of these two genome assemblies using LAST v.926 (Kiełbasa *et al.* 2011), which only considered the reliably aligned regions longer than 1 Mb (e-value ≤ 1e-5). The results were visualized using Circos v.0.96-9 (Krzywinski *et al.* 2009).

### Retrieval of the mitochondrial genome

The mitochondrial genome sequence was identified as a single scaffold by searching the available mitochondrial genome of the *S. glanis* (NC_014261.1, from Kastoria Lake, Greece; Vittas *et al.* 2011) against the generated genome assembly using NCBI’s blastn v.2.9.0 (Boratyn *et al.* 2013; evalue 1e-10, -soft_masking true, -lcase_masking, and a hit fraction filter to include only hits of > 90% target length, -qcov_hsp_perc 90). Further, both the retrieved and published (NC_014261.1) mitochondrial genomes were aligned, and genetic variants were identified. The genomic classification of genetic variants into different categories (synonymous, non-synonymous, exonic and intergenic) was performed using SnpEff v.4.3t (Cingolani *et al.* 2012).

### Transcriptome assembly

To assist the subsequent genome annotation, we performed RNA sequencing and *de novo* transcriptome assembly based on eleven tissues. A total of 364.0 M RNA-seq read-pairs were generated. Short (< 50 bp) and low-quality reads (average quality ≤ 25) were trimmed using Trimmomatic v.0.35 (Bolger *et al.* 2014); SLIDINGWINDOW:5:25 MINLEN:50). Erroneous k-mers were removed from the Illumina paired-end reads, and random sequencing errors were corrected using rCorrector (Song and Florea 2015). The corrected and trimmed reads were mapped to an rRNA database (SILVA Release 132; Pruesse *et al.* 2007) to further reduce bias in downstream analyses due to over ribo-depletion (Lahens *et al.* 2014). Finally, 318.5 M filtered read-pairs were assembled *de novo* using Trinity v.2.5.1 (Haas *et al.* 2013) with default parameters. The redundancy of the transcriptome assembly was reduced as described in Eccles *et al.* (2018). Briefly, the quality-controlled RNA-seq reads were mapped to the Trinity-generated transcriptome assembly using Salmon v.1.1.0 (Love *et al.* 2018). A threshold of 100 mapped reads in BUSCO genes was chosen, as 98% of complete BUSCO sequences had more than 100 mapped reads, retaining 80,005 of the initial 250,220 transcripts. The longest Met to Stop open reading frame was identified for each transcript for protein-based clustering with CD-HIT v.4.7 (Li and Godzik 2006; Fu *et al.* 2012) at 98% identity, retaining 56,266 transcripts. Finally, the longest isoform of each gene was identified, producing an isoform-collapsed transcriptome subset, which was clustered at the protein level by CD-HIT v.4.7 at 90% identity. This resulted in a final dataset of 48,141 transcripts.

### Repetitive-sequence discovery

First, a *de novo* repeat library was generated using RepeatModeler v.1.0.11 (Smit and Hubley 2008-2015) with default parameters. Further, RepeatMasker v.4.0.8 (Smit *et al*. 2013-2015) was applied to screen for repeats and low complexity regions in the assembly using the generated *de novo* repeat library in combination with Dfam consensus 20171107 (Hubley *et al.* 2016) and RepBase 20181026 (Bao *et al.* 2015) repeat libraries. In addition, putative repeat regions were identified using Red v. 05/22/2015, a repeat-detection tool applying machine learning, which is capable of labeling its training data and training itself automatically on an entire genome (Girgis 2015). Finally, a consensus bed-file containing repeat coordinates derived from both methods was merged using bedtools v.2.27.1 (Quinlan and Hall 2010).

### Gene prediction and annotation

Gene models were predicted with MAKER v 3.01.2-beta (Holt and Yandell 2011), which combines *ab initio* gene prediction, RNA-seq assisted prediction and homology-based prediction. Repetitive genomic regions of the *S. glanis* genome were masked based on the repeat annotation results. Three MAKER runs were performed. During the first run, the *S. glanis* transcripts and protein sequences of 75 other fish species from the Ensembl 99 database (January 2020) were aligned to the genome as evidence to retrain Augustus v.3.3.3 (Stanke *et al.* 2006) and SNAP v. 2013-11-29 (Korf 2004) *ab initio* gene prediction tools. Gene annotations generated in the first (and second) runs of *ab initio* gene prediction tools were used in the second and third runs of MAKER. The BUSCO v.3.0.2b pipeline was applied to retrain Augustus using genomic regions containing mRNA annotations from the first and second MAKER run (including additional 1,000 bp on each side). BUSCO runs were performed using the–long option to optimize the HMM settings of the raw zebrafish HMM (–sp zebrafish; first run) or trained *S. glanis* HMM (second run), and to generate the final trained *S. glanis* HMM. SNAP was retrained using gene models from the first and second MAKER run with an annotation edit distance (AED) ≤ 0.25 and a length of amino acids ≥ 50. The AED quantifier ranges from 0 to 1 and shows the match between a gene annotation and its supporting evidence (EST, protein and mRNA-seq alignments). Lower AED values indicate higher congruency between the intron-exon coordinates of an annotation and its aligned evidence, whereas AED = 1 indicates no evidence for support of predicted genes. Only sequences with AED < 0.5 and coding sequences (CDS) ≥ 90 bp were retained in the final set of predicted genes.

The completeness of the predicted gene transcripts was evaluated with BUSCO v.3.0.2b (Simão *et al.* 2015) using a database of ray-finned fishes (Actinopterygii obd9). In addition, the number of reads mapped to the predicted genes was estimated by mapping the quality-controlled RNA-seq reads to the predicted transcriptome using Salmon v.1.1.0 (Love *et al.* 2018).

Functional annotation of the predicted proteins against vertebrate protein sequences in NCBI’s non-redundant database was performed using NCBI’s blastp v.2.9.0 (Boratyn *et al.* 2013; -evalue 1e-10, -soft_masking true, -lcase_masking, and a hit fraction filter to include only hits of > 70% target length, -qcov_hsp_perc 70). Next, non-annotated sequences were searched against all the protein sequences in the NCBI non-redundant database. In addition, Interproscan v.5.30-69.0 (Jones *et al.* 2014) was applied to search for protein domains, motifs and signatures present in the predicted protein sequences by searching against publicly available databases, including PANTHER 12.0 (Thomas *et al.* 2003), Pfam v.31.0 (Finn *et al.* 2014), PRINTS v.42.0 (Attwood *et al.* 2012), PROSITE v.2018_02 (Sigrist *et al.* 2013), SFLD 3 (Akiva *et al.* 2014), SMART v.7.1 (Letunic *et al.* 2012), SUPERFAMILY v.1.75 (de Lima Morais *et al.* 2011), and TIGRFAM v.15.0 (Haft *et al.* 2013).

### Inference of demographic history

The pairwise sequentially Markovian coalescent (PSMC) method was applied to infer the demographic history of *S. glanis* from a diploid sequence using psmc v.0.6.5 (Li and Durbin 2011). In brief, the quality and barcode-trimmed Illumina reads were aligned to the *S. glanis* reference genome assembly using bowtie2 v.2.3.5.1 (Langmead and Salzberg 2012) applying default parameters, except the modified score minimum threshold (–score-min L,-0.3,-0.3) and the maximum fragment length for valid paired-end alignments (-X 700). SAMtools v.1.10 (Li 2011) pipeline was further applied to generate the diploid consensus sequences with default settings (https://github.com/lh3/psmc), except that the minimum and maximum read depths were set to 15 and 90, respectively (-d 15 -D 90) to obtain high quality SNP data. The input file for PSMC modeling was generated with the fq2psmcfa tool (-q 20) and processed in psmc applying the following parameters: -N25 -t15 -r5 -p ‘4+25*2+4+6’ (Liu *et al.* 2016). A generation time of 6 years (Froese and Pauly 2019) and a mutation rate of 2.5e-08 (Liu *et al.* 2016) were applied for time calibration.

### Data availability

Short Illumina linked-reads are available in the NCBI Sequence Read Archive (SRA; SSR11087275-SSR11087276 and SSR11087282-SSR11087283), and the Whole Genome Assembly has been deposited at DDBJ/EMBL/GenBank under the accession JAAIIK000000000, both under BioProject PRJNA605930. Transcriptome reads are available in the NCBI SRA (SRR11087269-SRR11087274 and SRR11087277-SRR11087281), and the Transcriptome Shotgun Assembly has been deposited at DDBJ/EMBL/GenBank under accession number GIPM00000000, as a part of BioProject PRJNA605930.

## Results And Discussion

### The S. glanis genome characteristics

Genome size estimates from GenomeScope ranged from 723.4 to 769.9 Mb, whereas estimates based on findGSE (fitted and original counts with corrected k-mer coverage) were higher, ranging from 822.6 Mb to 906.5 Mb (Table 1). The estimated genome size was comparable to the most closely related *S. asotus* (831 – 1,411 Mb, Jianxun *et al.* 1991) and other Siluriformes, whose genome sizes vary from 599 Mb in *B. yarrelli* (Jiang *et al.* 2019) to 1,200 Mb in *C. batrachus* (Li *et al.* 2018).

**Table 1:** The *S. glanis* genome size, heterozygosity and repeat content as estimated by the GenomeScope and findGSE software

Genome characteristics	k-mer size		
	k = 17	k = 21	k = 25
*GenomeScope*			
Genome haploid length (Mb)	723.4	753.6	769.9
Genome repeat length (Mb)	337.8	216.8	205.4
Genome unique length (Mb)	385.6	536.7	564.5
Heterozygosity, %	0.24	0.25	0.23
Estimated repetitive ratio,%	46.7	28.8	26.7
Read error rate, %	0.30	0.36	0.36
*findGSE*			
Genome haploid length (Mb)	822.6	901.3	906.5
Genome repeat length (Mb)	395.1	326.9	300.2
Genome unique length (Mb)	427.5	574.5	606.3
Estimated repetitive ratio, %	48.0	36.3	33.1

The GenomeScope analysis revealed low heterozygosity of the *S. glanis* genome (0.23 – 0.25%, Table 1) in comparison with other species (*e.g.*, Kajitani *et al.* 2014; Vurture *et al.* 2017). The estimated proportion of repetitive regions in the *S. glanis* genome was high, ranging from 26.7% (k = 25, GenomeScope) to 48.0% (k = 17, findGSE). These estimates are comparable to other Siluriformes, in which the proportion of repeats in the genome varies from 30% in *C. batrachus* (Li *et al.* 2018) to 44% in *I. punctatus* (Yuan *et al.* 2018).

### Genome assembly

The total length of the assembly was 793.4 Mb, which included ca. 10% (80.4 Mb) of unknown bases (Table 2). Such a high proportion of unknown bases is typical for genome assemblies generated using linked reads with 10X Chromium technology (*e.g.*, Mohr *et al.* 2017; Hulse-Kemp *et al.* 2018), as the Supernova assembler estimates gap sizes rather than introducing an arbitrary value of Ns during scaffolding (Weisenfeld *et al.* 2017). In the presented *S. glanis* genome assembly, repeat regions were estimated to account for 39.5% (313.3 Mb). The contig N50 and scaffold N50 sizes were 13.9 Kb and 3.2 Mb, respectively (Table 2). Altogether, 282 of the longest scaffolds (≥ 250 Kb; 1.1% of all scaffolds) covered more than 80% of the genome assembly.

**Table 2:** The *S. glanis* genome assembly and annotation statistics

	Genome assembly*^a^*
*Contig statistics*
Number of contigs	105,816
Total contig size (bp)	712,999,588
Contig *N*_50_ size (bp)	13,869
Largest contig (bp)	140,841
*Scaffold statistics*	
Number of scaffolds	25,703
Total scaffold size (bp)	793,358,859
Scaffold *N*_50_ size (bp)	3,169,562
Largest scaffold (bp)	13,715,129
GC content (%)	39.2
Unknown base (%)	10.1
*BUSCO genome completeness*	
Complete	3,859 (84.2%)
Complete and single copy	3,717 (81.1%)
Complete and duplicated	142 (3.1%)
Fragmented	312 (6.8%)
Missing	413 (9.0%)
*Annotation*	
Number of protein-coding genes	21,316
with partial EST support	10,260
with > 90% EST support	4,989
with full length EST support	3,795
with > 100 RNAseq reads aligned	17,330
with > 10 RNAseq reads aligned	19,855
Number of functionally-annotated proteins	20,532
Mean protein length (interquartile range, aa)	501 (218-617)
Longest protein (aa)	27,306 (titin-like)
Average number of exons per gene (mean length, interquartile range)	9 (212, 89-194 bp)
Average number of introns per gene (length, interquartile range)	8 (1,208, 133-1,274 bp)
*BUSCO completeness of the predicted gene models*	
Complete	3,427 (74.8%)
Complete and single copy	3,248 (70.9%)
Complete and duplicated	179 (3.9%)
Fragmented	403 (8.8%)
Missing	754 (16.4%)

a Minimum scaffold length: 1 Kb.

**Figure 2:**
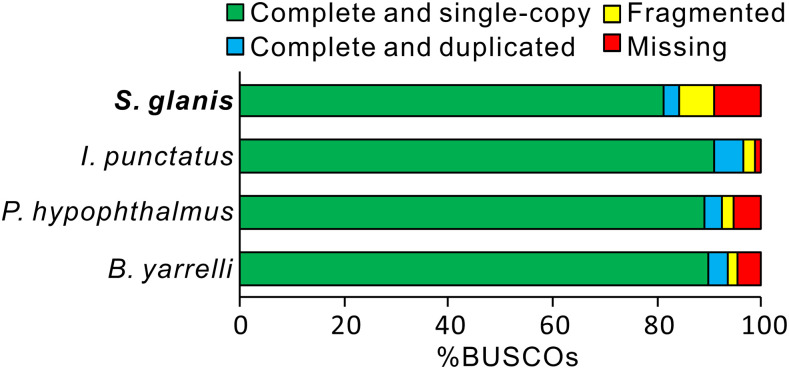
BUSCO assessment of the *S. glanis* and other Siluriformes genomes.

**Figure 3:**
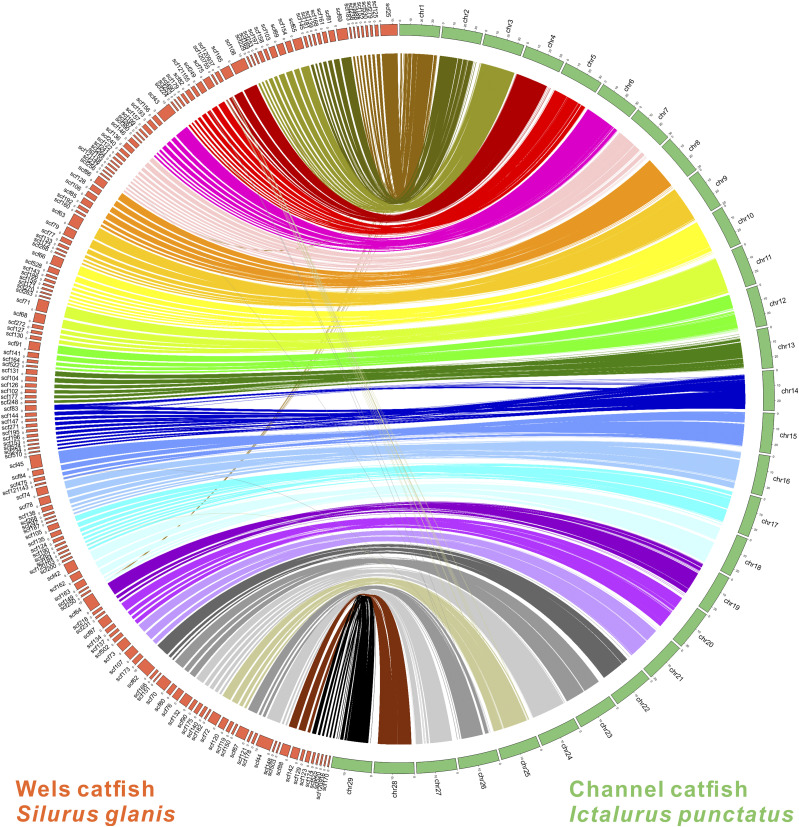
Circos plot showing the high level of synteny between *S. glanis* (this study) and *I. punctatus* (Liu *et al.* 2016).

The presented *S. glanis* genome assembly covered 84.2% complete and 6.8% partial ray-finned fishes BUSCOs (Table 2). Despite that the completeness of genome assemblies in other catfishes, generated with the assistance of long-read technologies, was ca. 5–10% higher (*e.g.*, Kim *et al.* 2018; Liu *et al.* 2016; Jiang *et al.* 2019) compared to the present genome assembly of *S. glanis* (Figure 2), this is comparable to other genome assemblies generated using only linked-read technology (*e.g.*, Hammond *et al.* 2017; Jones *et al.* 2017; Ozerov *et al.* 2018; Lu *et al.* 2020). In addition, high completeness and accuracy of the presented *S. glanis* genome assembly was supported by high sequence similarities and concordant alignment with the chromosome regions of *I. punctatus* (Liu *et al.* 2016; Figure 3).

### Transcriptome assembly

The *S. glanis* transcriptome assembly based on multiple tissues consisted of 48,133 transcripts covering 92.1% complete and 2.7% partial ray-finned fish BUSCOs. The total transcriptome and N50 transcript size were 80.8 Mb and 2.4 Kb, respectively (Table 3).

**Table 3:** The *S. glanis* transcriptome assembly statistics

	Transcriptome assembly (multiple tissues)
*Transcript statistics*	
Number of transcripts	48,133
Total transcript size (bp)	80,812,654
Transcript *N*_50_ size (bp)	2,394
Largest transcript (bp)	69,646
*BUSCO transcriptome completeness*	
Complete	4,222 (92.1%)
Complete and single copy	3,844 (83.9%)
Complete and duplicated	378 (8.2%)
Fragmented	123 (2.7%)
Missing	239 (5.2%)

### Genome annotation

In total 21,316 protein-coding genes were predicted in the present *S. glanis* genome with the MAKER annotation pipeline (Table 2). The putative function annotation based on homology with NCBI’s blastp was obtained for 19,627 proteins (92.1%). Further, domains, motifs and signatures were detected for 19,931 proteins (93.5%) with Interproscan. As a result, 20,532 genes were annotated by at least one of the two methods, accounting for approximately 96.3% of the gene models of *S. glanis* (Table 2).

### Mitochondrial genome

In addition to the nuclear genome, we successfully retrieved a nearly complete *S. glanis* mitochondrial genome consisting of 16,527 bp (scf113625_mtDNA). By comparing the newly assembled genome with the previously published mito-genome of *S. glanis* (NC_014261.1, Vittas *et al.* 2011), we detected 79 SNPs (of which 29 were non-synonymous) corresponding to 0.4% sequence divergence. Assuming a conventional mtDNA molecular clock of 2% sequence divergence per million years (Brown *et al.* 1979), this places the estimated time of divergence of these two lineages from a common ancestor to approximately 200,000 years ago. Such a deep divergence is consistent with the earlier work on mtDNA variation based on PCR-RFLP analysis of cytochrome b, control region and ND-5/6 regions in *S. glanis*, which have revealed two major evolutionary lineages in Europe (Krieg *et al.* 2000).

### Population history of S. glanis

**Figure 4:**
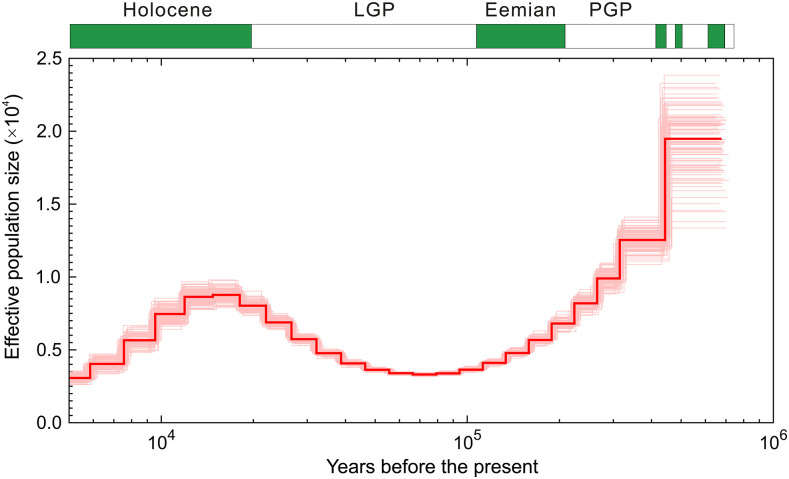
Inference of the *S. glanis* demographic history as revealed by PSMC analysis. The inferred population size is presented as a bold red line, and the surrounding thin pink lines are the estimates of population size generated after 100 rounds of bootstrapping. Green and white bars above the figure represent interglacial and glacial periods, respectively. The names of the last four geological epochs are indicated above the bars: the Holocene, the Last Glacial Period (LGP), the Eemian and the Penultimate Glacial Period (PGP).

Using the pairwise sequentially Markovian coalescent model (Li and Durbin 2011), we inferred the *S. glanis* historic population dynamics (Figure 4). The large effective population size of the *S. glanis* populations observed ca. 500,000 – 250,000 years ago declined to its minimum during the Last Glacial Period (ca. 115,000 – 11,700 years ago) with the following recovery in the earlier Holocene (ca. 11,650 – 8,000 years ago). Thereafter, the effective population size of *S. glanis* started to show another declining trend.

It should be noted that the estimates of historical fluctuations of the effective population size observed for *S. glanis* should be taken with caution, as the model itself estimates the rate of coalescence at each point in time, which in turn can be used as a proxy for population size under certain assumptions (Mather *et al.* 2020). Thus, there are many factors affecting the relationship between coalescence times and population size, such as natural selection and non-random mating (Mazet *et al.* 2016). Furthermore, when the migration rate between populations is low (*e.g.*, island model as an example of non-random mating; Wright 1931), the inferred coalescent effective population size may be overestimated (Li & Durbin 2011), leading to a reflection of increased population structure rather than increased population size. In other words, the estimates of the effective population size at the time of separation will be biased upwards with increased within-species genetic structure (Nadachowska-Brzyska *et al.* 2016). The pattern observed for *S. glanis* in our study may therefore indicate that within-species population structure remained at lower levels during the Eemian interglacial (130,000 – 115,000 years ago) compared to the earlier Holocene, due to a higher connectivity of water bodies during the former (Tudryn *et al.* 2016; Krijgsman *et al.* 2019). In the earlier Holocene, however, *S. glanis* recolonization of western Europe from the eastern refugium – presumably located around the Ponto-Caspian region (Krieg *et al.* 2000) – increased the within-species structuring as the ice sheet gradually retreated.

## Conclusions

We performed whole-genome assembly, using a combination of 10X Chromium linked-read technology and accurate short-read sequencing, to generate the first genome reference for *S. glanis*. More than 21,000 protein coding genes were identified; among these, 19,627 genes were annotated with known homology, which will facilitate further functional genomic and gene ontology analyses. The scaffold length (*N*_50_ = 3.2 Mb) of the generated genome assembly will facilitate the detection of genomic variants, including copy number variations and large insertions/deletions. Given the ecological, cultural and economic importance of *S. glanis*, as well as its increasing aquaculture production, the generated genome assembly will serve as a backbone for future genomics, genetics, conservation, and breeding research of *S. glanis* and other fish species of the Siluridae family.

## References

[bib1] Adamek Z., Grecu I., Metaxa I., Sabarich L., and Blancheton J. P., 2015 Processing traits of European catfish (*Silurus glanis* Linnaeus, 1758) from outdoor flow-through and indoor recycling aquaculture units. J. Appl. Ichthyology 31: 38–44. 10.1111/jai.12848

[bib2] Akiva E., Brown S., Almonacid D. E., Barber A. E. 2nd, Custer A. F., 2014 The structure-function linkage database. Nucleic Acids Res. 42: D521–D530. 10.1093/nar/gkt1130 24271399 PMC3965090

[bib3] Alp A., Kara C., Üçkardeş F., Carol J., and García-Berthou E., 2011 Age and growth of the European catfish (*Silurus glanis*) in a Turkish Reservoir and comparison with introduced populations. Rev. Fish Biol. Fish. 21: 283–294. 10.1007/s11160-010-9168-4

[bib4] Attwood T. K., Coletta A., Muirhead G., Pavlopoulou A., Philippou P. B., 2012 The PRINTS database: a fine-grained protein sequence annotation and analysis resource – its status in 2012. Database (Oxford) 2012: bas019 10.1093/database/bas019 22508994 PMC3326521

[bib5] Bao W., Kojima K. K., and Kohany O., 2015 Repbase Update, a database of repetitive elements in eukaryotic genomes. Mob. DNA 6: 11 10.1186/s13100-015-0041-9 26045719 PMC4455052

[bib6] Berg L. S., 1949 Freshwater fishes of the USSR and adjacent countries, Academy of Sciences of the USSR, Zoological Institute, Leningrad.

[bib7] Bolger A. M., Lohse M., and Usadel B., 2014 Trimmomatic: a flexible trimmer for Illumina sequence data. Bioinformatics 30: 2114–2120. 10.1093/bioinformatics/btu170 24695404 PMC4103590

[bib8] Boratyn G.M., Camacho C., Cooper P.S., Coulouris G., Fong A., 2013 BLAST: a more efficient report with usability improvements. Nucleic Acids Res. 41: W29–W33. 10.1093/nar/gkt282 23609542 PMC3692093

[bib9] Brown W. M., George M. Jr., and Wilson A. C., 1979 Rapid evolution of animal mitochondrial DNA. Proc. Natl. Acad. Sci. USA 76: 1967–1971. 10.1073/pnas.76.4.1967 109836 PMC383514

[bib10] Cingolani P., Platts A., Wang L. L., Coon M., Nguyen T., 2012 A program for annotating and predicting the effects of single nucleotide polymorphisms, SnpEff. Fly (Austin) 6: 80–92. 10.4161/fly.19695 22728672 PMC3679285

[bib11] Copp G. H., Robert Britton J., Cucherousset J., García-Berthou E., Kirk R., 2009 Voracious invader or benign feline? A review of the environmental biology of European catfish *Silurus glanis* in its native and introduced ranges. Fish Fish. 10: 252–282. 10.1111/j.1467-2979.2008.00321.x

[bib12] Cucherousset J., Horky P., Slavík O., Ovidio M., Arlinghaus R., 2018 Ecology, behaviour and management of the European catfish. Rev. Fish Biol. Fish. 28: 177–190. 10.1007/s11160-017-9507-9

[bib13] Jianxun C., Xiuhai R., Qixing Y., 1991 Nuclear DNA Content Variation in Fishes. Cytologia (Tokyo) 56: 425–429. 10.1508/cytologia.56.425

[bib14] de Lima Morais D. A., Fang H., Rackham O. J., Wilson D., Pethica R., 2011 SUPERFAMILY 1.75 including a domain-centric gene ontology method. Nucleic Acids Res. 39: D427–D434. 10.1093/nar/gkq1130 21062816 PMC3013712

[bib15] Eccles D., Chandler J., Camberis M., Henrissat B., Koren S., 2018 De novo assembly of the complex genome of *Nippostrongylus brasiliensis* using MinION long reads. BMC Biol. 16: 6 10.1186/s12915-017-0473-4 29325570 PMC5765664

[bib16] FAO, 2020. Fishery and Aquaculture Statistics. Global aquaculture production 1950–2018 (FishstatJ). In: FAO Fisheries and Aquaculture Department [online]. Rome. Updated 2020. www.fao.org/fishery/statistics/software/fishstatj/en.

[bib17] Finn R. D., Bateman A., Clements J., Coggill P., Eberhardt R. Y., 2014 Pfam: the protein families database. Nucleic Acids Res. 42: D222–D230. 10.1093/nar/gkt1223 24288371 PMC3965110

[bib18] Frimodt C., 1995 Multilingual illustrated guide to the world’s commercial coldwater fish, Fishing News Books Ltd., Oxford, UK.

[bib19] Froese, R., and D. Pauly. Editors. 2019 FishBase. World Wide Web electronic publication. www.fishbase.org, version (12/2019).

[bib20] Fu L., Niu B., Zhu Z., Wu S., and Li W., 2012 CD-HIT: accelerated for clustering the next-generation sequencing data. Bioinformatics 28: 3150–3152. 10.1093/bioinformatics/bts565 23060610 PMC3516142

[bib21] Girgis H. Z., 2015 Red: an intelligent, rapid, accurate tool for detecting repeats de-novo on the genomic scale. BMC Bioinformatics 16: 227 10.1186/s12859-015-0654-5 26206263 PMC4513396

[bib22] Gong G., Dan C., Xiao S., Guo W., Huang P., 2018 Chromosomal-level assembly of yellow catfish genome using third-generation DNA sequencing and Hi-C analysis. Gigascience 7: giy120. 10.1093/gigascience/giy120 PMC6228179 30256939

[bib23] Gremme G., Steinbiss S., and Kurtz S., 2013 GenomeTools: A comprehensive software library for efficient processing of structured genome annotations. IEEE/ACM Trans. Comput. Biol. Bioinformatics 10: 645–656. 10.1109/TCBB.2013.68 24091398

[bib24] Gurevich A., Saveliev V., Vyahhi N., and Tesler G., 2013 QUAST: quality assessment tool for genome assemblies. Bioinformatics 29: 1072–1075. 10.1093/bioinformatics/btt086 23422339 PMC3624806

[bib25] Haas B. J., Papanicolaou A., Yassour M., Grabherr M., Blood P. D., 2013 De novo transcript sequence reconstruction from RNA-seq using the Trinity platform for reference generation and analysis. Nat. Protoc. 8: 1494–1512. 10.1038/nprot.2013.084 23845962 PMC3875132

[bib26] Haft D. H., Selengut J. D., Richter R. A., Harkins D., Basu M. K., 2013 TIGRFAMs and genome properties in 2013. Nucleic Acids Res. 41: D387–D395. 10.1093/nar/gks1234 23197656 PMC3531188

[bib27] Hammond S. A., Warren R. L., Vandervalk B. P., Kucuk E., Khan H., 2017 The North American bullfrog draft genome provides insight into hormonal regulation of long noncoding RNA. Nat. Commun. 8: 1433 10.1038/s41467-017-01316-7 29127278 PMC5681567

[bib28] Holt C., and Yandell M., 2011 MAKER2: an annotation pipeline and genome-database management tool for second-generation genome projects. BMC Bioinformatics 12: 491 10.1186/1471-2105-12-491 22192575 PMC3280279

[bib29] Howe K., Clark M. D., Torroja C. F., Torrance J., Berthelot C., 2013 The zebrafish reference genome sequence and its relationship to the human genome. Nature 496: 498–503. 10.1038/nature12111 23594743 PMC3703927

[bib30] Hubley R., Finn R. D., Clements J., Eddy S. R., Jones T. A., 2016 The Dfam database of repetitive DNA families. Nucleic Acids Res. 44: D81–D89. 10.1093/nar/gkv1272 26612867 PMC4702899

[bib31] Hulse-Kemp A. M., Maheshwari S., Stoffel K., Hill T. A., Jaffe D., 2018 Reference quality assembly of the 3.5-Gb genome of Capsicum annuum from a single linked-read library. Hortic. Res. 5: 4 10.1038/s41438-017-0011-0 29423234 PMC5798813

[bib32] Jackson A. P., Sanders M., Berry A., McQuillan J., Aslett M. A., 2010 The genome sequence of *Trypanosoma brucei gambiense*, causative agent of chronic human African trypanosomiasis. PLoS Negl. Trop. Dis. 4: e658 10.1371/journal.pntd.0000658 20404998 PMC2854126

[bib33] Jankowska B., Zakęś Z., Żmijewski T., Ulikowski D., and Kowalska A., 2006 Slaughter value and flesh characteristics of European catfish (*Silurus glanis*) fed natural and formulated feed under different rearing conditions. Eur. Food Res. Technol. 224: 453–459. 10.1007/s00217-006-0349-2

[bib34] Jiang W., Lv Y., Cheng L., Yang K., Bian C., 2019 Whole-genome sequencing of the giant devil catfish, *Bagarius yarrelli*. Genome Biol. Evol. 11: 2071–2077. 10.1093/gbe/evz143 31274158 PMC6681832

[bib35] Jones P., Binns D., Chang H. Y., Fraser M., Li W., 2014 InterProScan 5: genome-scale protein function classification. Bioinformatics 30: 1236–1240. 10.1093/bioinformatics/btu031 24451626 PMC3998142

[bib36] Jones S. J. M., Taylor G. A., Chan S., Warren R. L., Hammond S. A., 2017 The genome of the beluga whale (*Delphinapterus leucas*). Genes (Basel) 8: 378 10.3390/genes8120378 PMC5748696 29232881

[bib37] Kai W., Kikuchi K., Tohari S., Chew A. K., Tay A., 2011 Integration of the genetic map and genome assembly of fugu facilitates insights into distinct features of genome evolution in teleosts and mammals. Genome Biol. Evol. 3: 424–442. 10.1093/gbe/evr041 21551351 PMC5654407

[bib38] Kajitani R., Toshimoto K., Noguchi H., Toyoda A., Ogura Y., 2014 Efficient de novo assembly of highly heterozygous genomes from whole-genome shotgun short reads. Genome Res. 24: 1384–1395. 10.1101/gr.170720.113 24755901 PMC4120091

[bib39] Kappas I., Vittas S., Pantzartzi C. N., Drosopoulou E., and Scouras Z. G., 2016 A Time-calibrated mitogenome phylogeny of catfish (Teleostei: Siluriformes). PLoS One 11: e0166988 10.1371/journal.pone.0166988 27907107 PMC5132296

[bib40] Kiełbasa S. M., Wan R., Sato K., Horton P., and Frith M. C., 2011 Adaptive seeds tame genomic sequence comparison. Genome Res. 21: 487–493. 10.1101/gr.113985.110 21209072 PMC3044862

[bib41] Kim O. T. P., Nguyen P. T., Shoguchi E., Hisata K., Vo T. T. B., 2018 A draft genome of the striped catfish, *Pangasianodon hypophthalmus*, for comparative analysis of genes relevant to development and a resource for aquaculture improvement. BMC Genomics 19: 733 10.1186/s12864-018-5079-x 30290758 PMC6173838

[bib42] Korf I., 2004 Gene finding in novel genomes. BMC Bioinformatics 5: 59 10.1186/1471-2105-5-59 15144565 PMC421630

[bib43] Kottelat, M., and J. Freyhof, 2007 *Handbook of European freshwater fishes*. Publications Kottelat, Cornol, Switzerland.

[bib44] Krieg F., Triantafyllidis A., and Guyomard R., 2000 Mitochondrial DNA variation in European populations of *Silurus glanis*. J. Fish Biol. 56: 713–724. 10.1111/j.1095-8649.2000.tb00767.x

[bib45] Krijgsman W., Tesakov A., Yanina T., Lazarev S., Danukalova G., 2019 Quaternary time scales for the Pontocaspian domain: Interbasinal connectivity and faunal evolution. Earth Sci. Rev. 188: 1–40. 10.1016/j.earscirev.2018.10.013

[bib46] Krzywinski M., Schein J., Birol I., Connors J., Gascoyne R., 2009 Circos: an information aesthetic for comparative genomics. Genome Res. 19: 1639–1645. 10.1101/gr.092759.109 19541911 PMC2752132

[bib47] Lahens N. F., Kavakli I. H., Zhang R., Hayer K., Black M. B., 2014 IVT-seq reveals extreme bias in RNA sequencing. Genome Biol. 15: R86 10.1186/gb-2014-15-6-r86 24981968 PMC4197826

[bib48] Langmead B., and Salzberg S. L., 2012 Fast gapped-read alignment with Bowtie 2. Nat. Methods 9: 357–359. 10.1038/nmeth.1923 22388286 PMC3322381

[bib49] Letunic I., Doerks T., and Bork P., 2012 SMART 7: recent updates to the protein domain annotation resource. Nucleic Acids Res. 40: D302–D305. 10.1093/nar/gkr931 22053084 PMC3245027

[bib50] Li H., 2011 A statistical framework for SNP calling, mutation discovery, association mapping and population genetical parameter estimation from sequencing data. Bioinformatics 27: 2987–2993. 10.1093/bioinformatics/btr509 21903627 PMC3198575

[bib51] Li H., and Durbin R., 2011 Inference of human population history from individual whole-genome sequences. Nature 475: 493–496. 10.1038/nature10231 21753753 PMC3154645

[bib52] Li N., Bao L., Zhou T., Yuan Z., Liu S., 2018 Genome sequence of walking catfish (*Clarias batrachus*) provides insights into terrestrial adaptation. BMC Genomics 19: 952 10.1186/s12864-018-5355-9 30572844 PMC6302426

[bib53] Li W., and Godzik A., 2006 Cd-hit: a fast program for clustering and comparing large sets of protein or nucleotide sequences. Bioinformatics 22: 1658–1659. 10.1093/bioinformatics/btl158 16731699

[bib54] Linhart O., Štěch L., Švarc J., Rodina M., Audebert J. P., 2002 The culture of the European catfish, *Silurus glanis*, in the Czech Republic and in France. Aquat. Living Resour. 15: 139–144. 10.1016/S0990-7440(02)01153-1

[bib55] Liu Z., Liu S., Yao J., Bao L., Zhang J., 2016 The channel catfish genome sequence provides insights into the evolution of scale formation in teleosts. Nat. Commun. 7: 11757 10.1038/ncomms11757 27249958 PMC4895719

[bib56] Love M. I., Soneson C., and Patro R., 2018 Swimming downstream: statistical analysis of differential transcript usage following Salmon quantification. F1000 Res. 7: 952 10.12688/f1000research.15398.1 PMC6178912 30356428

[bib57] Lu L., Zhao J., and Li C., 2020 High-quality genome assembly and annotation of the big-eye mandarin fish (*Siniperca knerii*). *G3 (Bethesda)*-. Genes Genom. Genet. 10: 877. 10.1534/g3.119.400930 PMC7056987 31953307

[bib58] Marçais G., and Kingsford C., 2011 A fast, lock-free approach for efficient parallel counting of occurrences of k-mers. Bioinformatics 27: 764–770. 10.1093/bioinformatics/btr011 21217122 PMC3051319

[bib59] Mather N., Traves S. M., and Ho S. Y. W., 2020 A practical introduction to sequentially Markovian coalescent methods for estimating demographic history from genomic data. Ecol. Evol. 10: 579–589. 10.1002/ece3.5888 31988743 PMC6972798

[bib60] Mazet O., Rodríguez W., Grusea S., Boitard S., and Chikhi L., 2016 On the importance of being structured: instantaneous coalescence rates and human evolution – lessons for ancestral population size inference? Heredity 116: 362–371. 10.1038/hdy.2015.104 26647653 PMC4806692

[bib61] Mohr, D.W., A. Naguib, N. Weisenfeld, V. Kumar, P. Shah *et al.*, 2017 Improved de novo genome assembly: Linked-read sequencing combined with optical mapping produce a high quality mammalian genome at relatively low cost. bioRxiv: 128348. (Preprint posted April 19, 2017) https://doi/org/10.1101/128348

[bib62] Nadachowska-Brzyska K., Burri R., Smeds L., and Ellegren H., 2016 PSMC analysis of effective population sizes in molecular ecology and its application to black-and-white Ficedula flycatchers. Mol. Ecol. 25: 1058–1072. 10.1111/mec.13540 26797914 PMC4793928

[bib63] Ozerov M. Y., Ahmad F., Gross R., Pukk L., Kahar S., 2018 Highly continuous genome assembly of Eurasian perch (*Perca fluviatilis*) using linked-read sequencing. *G3 (Bethesda)*-. Genes Genom. Genet. 8: 3737–3743. 10.1534/g3.118.200768 PMC6288837 30355765

[bib64] Pruesse E., Quast C., Knittel K., Fuchs B. M., Ludwig W., 2007 SILVA: a comprehensive online resource for quality checked and aligned ribosomal RNA sequence data compatible with ARB. Nucleic Acids Res. 35: 7188–7196. 10.1093/nar/gkm864 17947321 PMC2175337

[bib65] Pruszynski T., and Pistelok F., 1999 Biological and economical evaluation of African and European catfish rearing in water recirculating systems. Arch. Pol. Fisheries 7: 343–352.

[bib66] Quinlan A. R., and Hall I. M., 2010 BEDTools: a flexible suite of utilities for comparing genomic features. Bioinformatics 26: 841–842. 10.1093/bioinformatics/btq033 20110278 PMC2832824

[bib67] Rondeau E. B., Minkley D. R., Leong J. S., Messmer A. M., Jantzen J. R., 2014 The genome and linkage map of the northern pike (*Esox lucius*): Conserved synteny revealed between the salmonid sister group and the Neoteleostei. PLoS One 9: e102089 10.1371/journal.pone.0102089 25069045 PMC4113312

[bib68] Sigrist C. J., de Castro E., Cerutti L., Cuche B. A., Hulo N., 2013 New and continuing developments at PROSITE. Nucleic Acids Res. 41: D344–D347. 10.1093/nar/gks1067 23161676 PMC3531220

[bib69] Simão F. A., Waterhouse R. M., Ioannidis P., Kriventseva E. V., and Zdobnov E. M., 2015 BUSCO: assessing genome assembly and annotation completeness with single-copy orthologs. Bioinformatics 31: 3210–3212. 10.1093/bioinformatics/btv351 26059717

[bib70] Smit, A. F. A., and R. Hubley, 2008–2015 RepeatModeler Open-1.0. Available at: http://www.repeatmasker.org. Accessed: June 30, 2019.

[bib86] Smit A. F. A., R. Hubley, P. Green, 2013–2015 RepeatMasker Open-4.0. Available at: http://www.repeatmasker.org. Accessed: June 30, 2019.

[bib71] Song L., and Florea L., 2015 Rcorrector: efficient and accurate error correction for Illumina RNA-seq reads. Gigascience 4: 48 10.1186/s13742-015-0089-y 26500767 PMC4615873

[bib72] Stanke M., Schöffmann O., Morgenstern B., and Waack S., 2006 Gene prediction in eukaryotes with a generalized hidden Markov model that uses hints from external sources. BMC Bioinformatics 7: 62 10.1186/1471-2105-7-62 16469098 PMC1409804

[bib73] Sun H., Ding J., Piednoel M., and Schneeberger K., 2018 findGSE: estimating genome size variation within human and Arabidopsis using k-mer frequencies. Bioinformatics 34: 550–557. 10.1093/bioinformatics/btx637 29444236

[bib74] Thomas P. D., Kejariwal A., Campbell M. J., Mi H., Diemer K., 2003 PANTHER: a browsable database of gene products organized by biological function, using curated protein family and subfamily classification. Nucleic Acids Res. 31: 334–341. 10.1093/nar/gkg115 12520017 PMC165562

[bib75] Tudryn A., Leroy S. A. G., Toucanne S., Gibert-Brunet E., Tucholka P., 2016 The Ponto-Caspian basin as a final trap for southeastern Scandinavian Ice-Sheet meltwater. Quat. Sci. Rev. 148: 29–43. 10.1016/j.quascirev.2016.06.019

[bib76] Vejřík L., Vejříková I., Blabolil P., Eloranta A. P., Kočvara L., 2017 European catfish (*Silurus glanis*) as a freshwater apex predator drives ecosystem via its diet adaptability. Sci. Rep. 7: 15970 10.1038/s41598-017-16169-9 29162872 PMC5698325

[bib77] Vij S., Kuhl H., Kuznetsova I. S., Komissarov A., Yurchenko A. A., 2016 Chromosomal-level assembly of the Asian seabass genome using long sequence reads and multi-layered scaffolding. PLoS Genet. 12: e1005954 10.1371/journal.pgen.1005954 27082250 PMC4833346

[bib78] Vittas S., Drosopoulou E., Kappas I., Pantzartzi C. N., and Scouras Z. G., 2011 The mitochondrial genome of the European catfish *Silurus glanis* (Siluriformes, Siluridae). J. Biol. Res. (Thessalon.) 15: 25–35.

[bib79] Vurture G. W., Sedlazeck F. J., Nattestad M., Underwood C. J., Fang H., 2017 GenomeScope: fast reference-free genome profiling from short reads. Bioinformatics 33: 2202–2204. 10.1093/bioinformatics/btx153 28369201 PMC5870704

[bib80] Weisenfeld N. I., Kumar V., Shah P., Church D. M., and Jaffe D. B., 2017 Direct determination of diploid genome sequences. Genome Res. 27: 757–767. 10.1101/gr.214874.116 28381613 PMC5411770

[bib81] Wood D. E., and Salzberg S. L., 2014 Kraken: ultrafast metagenomic sequence classification using exact alignments. Genome Biol. 15: R46 10.1186/gb-2014-15-3-r46 24580807 PMC4053813

[bib82] Wright S., 1931 Evolution in Mendelian populations. Genetics 16: 97–159. 17246615 10.1093/genetics/16.2.97 PMC1201091

[bib83] Yuan Z., Zhou T., Bao L., Liu S., Shi H., 2018 The annotation of repetitive elements in the genome of channel catfish (Ictalurus punctatus). PLoS One 13: e0197371 10.1371/journal.pone.0197371 29763462 PMC5953449

[bib84] Zhang S., Li J., Qin Q., Liu W., Bian C., 2018 Whole-genome sequencing of Chinese yellow catfish provides a valuable genetic resource for high-throughput identification of toxin genes. Toxins (Basel) 10: 488 10.3390/toxins10120488 PMC6316204 30477130

[bib85] Zheng G. X. Y., Lau B. T., Schnall-Levin M., Jarosz M., Bell J. M., 2016 Haplotyping germline and cancer genomes with high-throughput linked-read sequencing. Nat. Biotechnol. 34: 303–311. 10.1038/nbt.3432 26829319 PMC4786454

